# Curious Case of Cobaltocenium Carbaldehyde

**DOI:** 10.1021/acs.organomet.2c00613

**Published:** 2023-02-21

**Authors:** Daniel Menia, Michael Pittracher, Holger Kopacka, Klaus Wurst, Florian R. Neururer, Daniel Leitner, Stephan Hohloch, Maren Podewitz, Benno Bildstein

**Affiliations:** † Institut für Allgemeine, Anorganische und Theoretische Chemie, Universität Innsbruck, Innrain 80-82, 6020 Innsbruck, Austria; ‡ Institute of Materials Chemistry, TU Wien, Getreidemarkt 9, 1060 Vienna, Austria

## Abstract

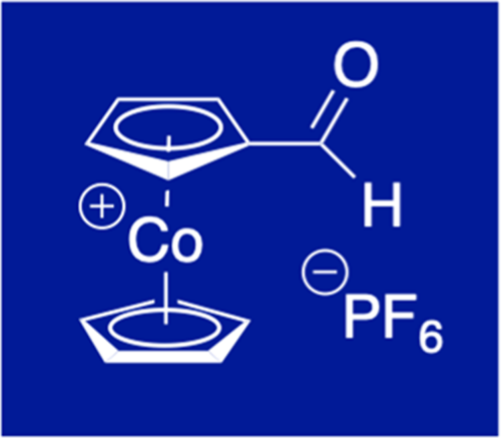

Cobaltocenium carbaldehyde (formylcobaltocenium) hexafluoridophosphate, a long sought-after functionalized cobaltocenium salt, is accessible from cobaltocenium carboxylic acid by a three-step synthetic sequence involving (i) chlorination to the acid chloride, (ii) copper-borohydride reduction to the hydroxymethyl derivative, and (iii) Dess–Martin oxidation to the title compound. Due to the strongly electron-withdrawing cationic cobaltocenium moiety, no standard aldehyde reactivity is observed. Instead, nucleophilic addition followed by haloform-type cleavage prevails, thereby ruling out common useful aldehyde derivatization. One-electron reduction of cobaltocenium carbaldehyde hexafluoridophosphate affords the deep-blue, isolable cobaltocene carbaldehyde 19-valence-electron radical whose spin density is located fully at cobalt and not at the formyl carbon atom. ^1^H/^13^C NMR, IR, EPR spectroscopy, high-resolution mass spectrometry, cyclic voltammetry, single crystal structure analysis (XRD), and density functional theory are applied to characterize these unusual formyl-cobaltocenium/cobaltocene compounds.

## Introduction

In the last couple of years, we have explored the chemistry of functionalized cobaltocenium salts^1^ aiming at developing the rather neglected chemistry of cobaltocenium compounds further in comparison to the very well researched and studied ferrocene derivatives and materials.^2^ Cobaltocenium salts are well worth to be investigated, due to their high chemical stability, fully reversible redox chemistry, high polarity with concomitant high solubility in polar solvents, and opposite electronic character (cationic charge, electron-withdrawing) to ferrocenes (neutral, electron-donating). To exploit these properties in more complex molecular compounds or materials, we need useful functionalized cobaltocenium synthons. So far, monofunctionalized cobaltocenium salts containing CO_2_H, CO_2_Cl, C≡CH, N_2_^+^, N_3_, I, Br, Cl, NH_2_, and Se–functionalities^1^ as well as O-triflate^3^ proved accessible and were used by us and others in diverse applications like redox catalysis,^1b,1d,1e^ bioorganometallic chemistry,^1a,1g,1h,4,5^ and redox-responsive macromolecules.^6^ A hitherto missing and long sought-after, synthetically highly promising functionalized cobaltocenium species is cobaltocenium carbaldehyde hexafluoridophosphate whose synthesis and peculiar properties are reported in this contribution.

## Results and Discussion

### Synthesis

**Scheme 1:**
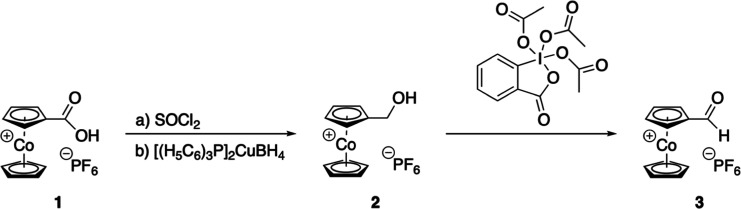
Synthesis of Cobaltocenium Carbaldehyde Hexafluoridophosphate (**3**)

Our first attempt to get access to a formylcobaltocenium salt dates back to 1998 when we tried to prepare pentamethylcobaltocene aldehyde by a half-sandwich capping reaction of [Cp*CoBr]_2_ with formylcyclopentadienide.^7^ However, this Co(II) aldehyde proved unstable and dimerized to the corresponding biscobaltocenium pinacol via a redox disproportionation of an in equilibrium present zwitterionic pentamethylcobaltocenium formyl radical anion.^7^ So far, cobaltocenium carbaldehyde remains unknown, but pentamethyliridocenium carbaldehyde hexafluoridophosphate has been briefly mentioned as a hydrolysis product from the corresponding oxazoline precursor.^8^ Having gained much experience in cobaltocenium chemistry in the last few years,^1^ we thought that reduction of cobaltocenium carboxylic acid hexafluoridophosphate^1i^ by a suitable reagent might provide a feasible synthetic route to a cobaltocenium aldehyde. However, most standard reducing agents like NaBH_4_, LiAlH_4_, or iBu_2_AlH are not applicable here, due to competing nucleophilic attack of hydride at the cationic cobaltocenium moiety.^9^ After screening of various reducing agents and reaction conditions, the following three-step procedure proved successful (Scheme 1). First, cobaltocenium carboxylic acid hexafluoridophosphate (**1**) was converted in neat thionyl chloride to its acid chloride,^1i^ followed by reduction with bis(triphenylphosphine)copper(I) tetrahydridoborate.^10^ This is considered in standard organic chemistry a selective reducing reagent for conversion of acid chlorides to aldehydes,^10^ but in our case, “over-reduction” to primary alcohol **2** was observed. In addition, and synthetically also important, no undesired nucleophilic attack of hydride at the cationic cobaltocenium moiety was evident. Hydroxymethylcobaltocenium hexafluoridophosphate (**2**) is a rather labile compound with limited shelf life; therefore it was just characterized by ^1^H NMR spectroscopy and then immediately used for the final oxidation step. With Dess-Martin periodinane,^11^**2** was oxidized to cobaltocenium carbaldehyde hexafluoridophosphate (**3**) in 38% yield from cobaltocenium carboxylic acid hexafluoridophosphate (**1**) over all three steps. Other common alcohol-aldehyde oxidations like Swern, Albright-Goldman, or Pfitzner-Moffatt activated DMSO reactions proved less suitable. Overall, we see that functional group transformations of cobaltocenium compounds are different compared to those of purely organic compounds, due to the directly attached, cationic, electron-withdrawing cobaltocenium moiety.

### Structural and Spectroscopic Properties

**Figure 1:**
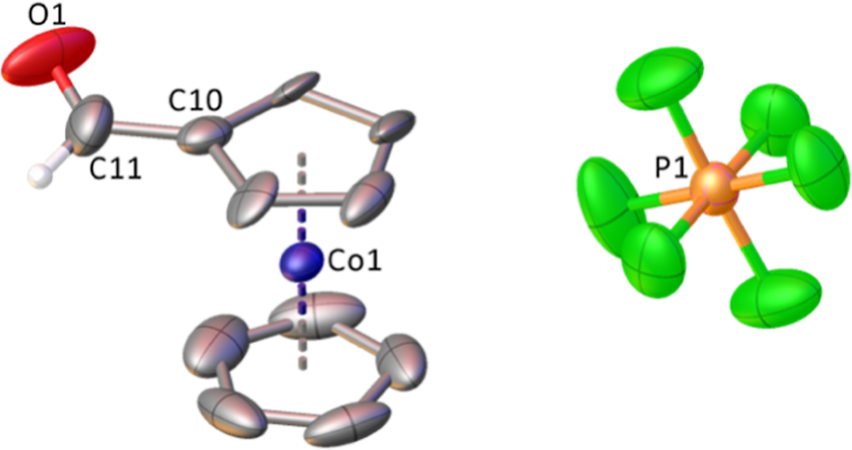
Molecular structure of **3**. Hydrogens except for the aldehyde proton omitted for clarity. Thermal ellipsoids are shown with 50% probability. Selected bond distances (Å): C(11)–O(1) = 1.222(9), C(10)–C(11) = 1.408(12), average value Co(1)–C(Cp) = 2.020.

Cationic aldehyde **3** has spectral properties of an acceptor-substituted aldehyde. The formyl group is clearly evident from its strong IR band observed at 1702 cm^–1^, and the hexafluoridophosphate counter ion gives rise to distinctive IR absorptions at 816 and 555 cm^–1^. ^1^H NMR data show a regular monosubstituted cobaltocenium moiety with the common pattern of two pseudo-triplets (δ = 6.42, 6.16 ppm) for the substituted Cp and a more intense singlet (δ = 6.05 ppm) for the unsubstituted Cp ligand. The aldehyde proton is observed at 10.18 ppm in the typical low field spectral region of aldehydes. Corresponding ^13^C chemical shifts are 189.6 ppm for the aldehyde carbon and 95.0–85.8 ppm for the Cp carbons. The identity of **3** is further corroborated by high-resolution mass spectrometry in agreement of the experimental most abundant monoisotopic peak and the calculated value. **3** is a yellow, moderately air-sensitive solid with a high melting point of 223.5 °C, in line with its ionic character. A single crystal structure analysis provides further unambiguous proof for the identity of **3** (Figure 1, Supporting Information).

### Reactivity

**Scheme 2:**
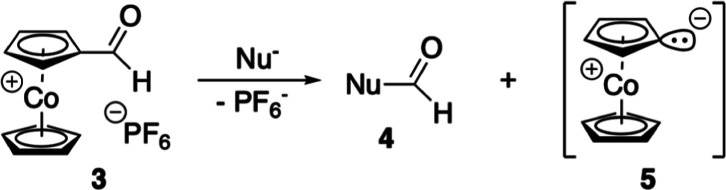
Nucleophilic Cleavage of Cobaltocenium Carbaldehyde Hexafluoridophosphate (**3**)

In organic chemistry, aldehydes are very useful synthons for a wide range of condensation and nucleophilic addition reactions. It is quite clear that a cationic aldehyde like **3** containing a directly attached electron-withdrawing cobaltocenium group will be much more reactive in comparison to organic aldehydes. This proved to be the case, although not in the anticipated manner. In contrast to our expectations, no successful condensation reactions (for example, Schiff base formation with aromatic primary amines or cyclocondensation with pyrrole to porphyrins) were possible; instead, degradation of the cobaltocenium aldehyde **3** under loss of the formyl group to parent, unsubstituted cobaltocenium hexafluorido-phosphate was observed. Chemically, this can be explained by a haloform-type cleavage^12^ of **3** under formation of formic derivative **4** and mesoionic cobaltocenylidene^13^**5** which easily gets protonated by solvent due to its high basicity (p*K*_a_ calculated = 38.5)^13^ (Scheme 2). In comparison to the classical haloform reaction of chloral with aqueous alkali to give chloroform and formic acid, cobaltocenium aldehyde **3** is obviously a (much) more reactive species that is easily cleaved even by nucleophiles of medium strength.

In an alternative interpretation, this reaction might be seen as a simple nucleophilic addition–nucleofuge elimination process. However, such reactions are usually favored with substrates containing good leaving groups such as halides or triflates that have a low basicity. In comparison to the leaving group CCl_3_^–^ (p*K*_a_ of CHCl_3_ = 15.5), cobaltocenylidene **5** (p*K*_a_ calculated = 38.5)^13^ is obviously an extremely poor leaving group.

The observed facile cleavage of cobaltocenium aldehyde **3** by nucleophiles rules out another interesting alternative reactivity of **3**: the potential deprotonation of **3** by suitable strong bases to a cobalt(I) ketene complex (η^4^-C_5_H_4_=C=O)Co(η^5^-C_5_H_5_) is clearly prevented by its high susceptibility to nucleophilic attack and subsequent cleavage as depicted in Scheme 2.

To gain some insights on the degree of electrophilic activation of the formyl carbon by the adjacent cationic cobaltocenium moiety, the group electronegativity (gEN)^14^ of the cobaltoceniumyl substituent (Cc^+^ = [C_10_H_9_Co]^+^) was calculated by density functional theory and compared to those of other electron-withdrawing substituents (Table 1). The second column lists gEN values according to ref (14a), while columns 3 and 4 denote gEN according to Allen’s electronegativity scale based on bond polarity indices when calculated with DFT (ωB97XD3(BJ)/def2-TZVP, column 3) and Hartree-Fock (UHF/6-31G*, column 4).^14c^ Column 5 lists gEN values based on Mulliken’s electronegativity scale, where it is defined as half the difference between electron affinity and ionization potential,^14d^ which was extended by De Proft.^14e^ As can be seen from inspection of Table 1, depending on the gEN scale, cobaltoceniumyl (Cc^+^) is a much more electronegative substituent in comparison to trichloromethyl and even more electronegative than pentafluorophenyl (except for the ωB97XD3(BJ)/def2-TZVP functional, column 5). It is interesting to note that the reactivity of pentafluorobenzaldehyde^15^ includes haloform-type cleavage, imine formation, and (cyclo)condensation reactions,^15^ in contrast to cobaltocenium carbaldehyde (**3**) where only haloform-type cleavage is observed, but no condensation reaction.

**Table 1:** Group Electronegativity (gEN) of Cobaltoceniumyl (Cc^+^) and Selected Electron-Withdrawing Substituents, Calculated with Various Methods^a^

substituent	reference (14a)^b^	reference (14c)^c^	reference (14c)^d^	reference (14d)^e^
CCl_3_	3.11	3.15	3.61	2.48
CF_3_	3.11	3.74	4.01	2.66
C(O)H	3.13	3.02	3.39	2.06
CN	3.19	2.98	3.30	3.87
NH_2_	3.22	2.80	3.21	2.62
C_6_F_5_	3.24	2.96	3.36	2.92
Cc^+^	3.26	3.55	3.90	4.33
NO_2_	3.75	3.89	4.48	2.90

a values are given in pauling units.

b B3LYP/aug-cc-pVTZ.

c ωB97XD3(BJ)/def2-TZVP.

d UHF/6-31G*.

e ωB97XD3(BJ)/def2-TZVP. Cc^+^ = [C_10_H_9_Co]^+^ Cobaltoceniumyl.

On the other hand, the intermediate generation of cobaltocenylidene (**5**) during the cleavage of cobaltocenium carbaldehyde (**3**) with nucleophiles like amines and pyrrole (Scheme 2) prompted us to attempt trapping cobaltocenylidene (**5**) with a suitable metal electrophile in the course of this reaction. Toward this desirable goal, some coinage metal precursors like Ag_2_O, Cu_2_O, [CuO^t^Bu]_4_, CuI, [Cu(CN)_2_]^−^, [Ag(CN)_2_],^–^ and [Au(CN)_2_]^−^ were reacted with **3** in the presence of nucleophiles like ^*t*^BuO^–^ or CN^–^ to effect cleavage and cobaltocenylidene metal complex formation. According to ^1^H NMR analysis, some at first promising results were obtained with copper(I) electrophiles but despite many attempts, we could not develop this reaction into a synthetically useful procedure.

### Redox Chemistry

**Figure 2:**
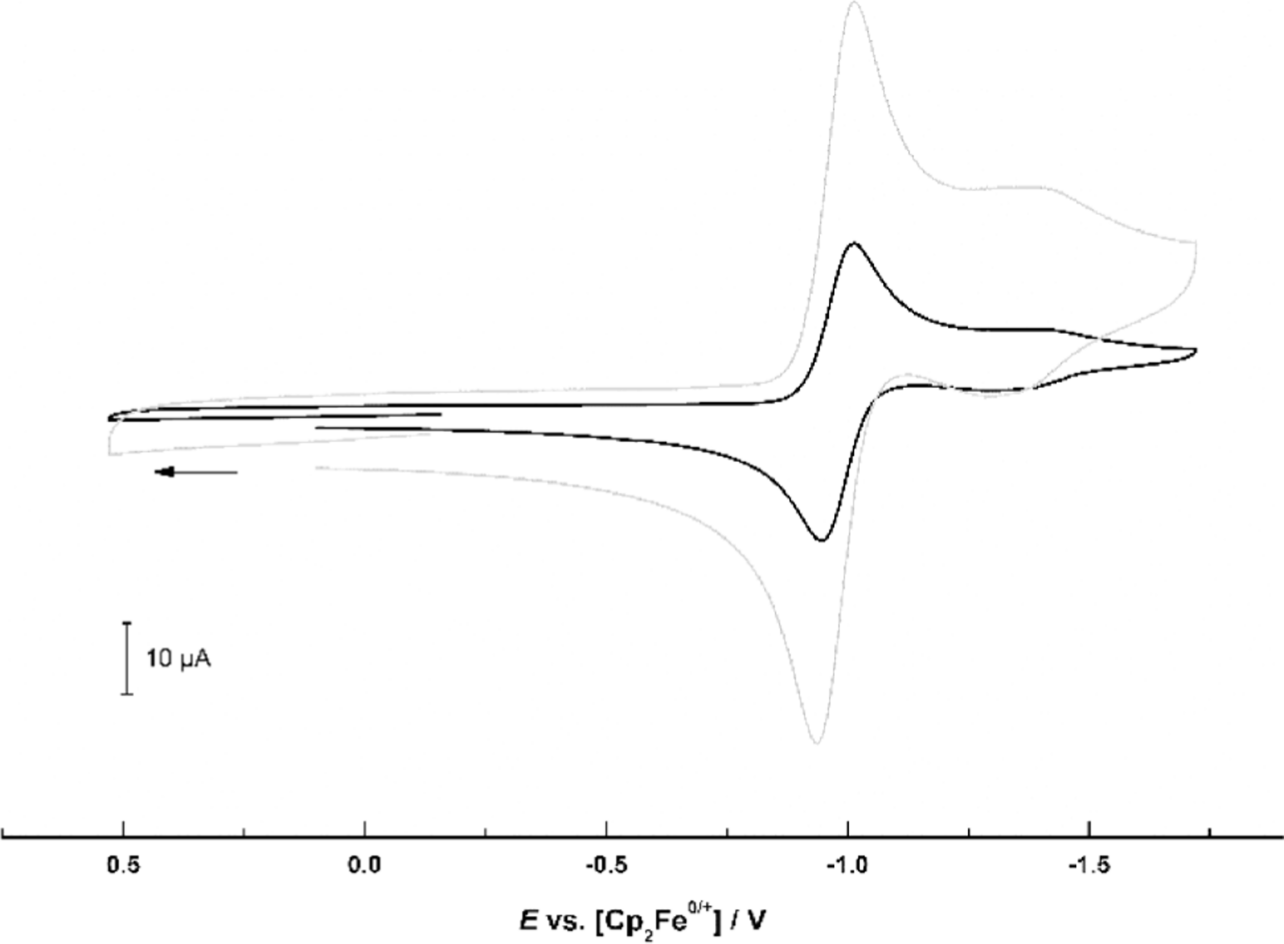
Cyclic voltammogram of cobaltocenium carbaldehyde hexafluoridophosphate (**3**) in CH_3_CN (0.15 M NBu_4_^+^PF_6_^–^) on a glassy carbon working electrode at sweep rates of 0.1 and 0.6 V s^–1^.

Cyclic voltammetry of cobaltocenium carbaldehyde hexafluoridophosphate (**3**) in acetonitrile solution referenced versus the ferrocene/ferrocenium couple (Figure 2) revealed a first reversible reduction (*E*_1/2_ = −0.98 V) followed by a second less intense wave (*E*_p_ = −1.42 V). For comparison, unsubstituted cobaltocenium hexafluoridophosphate has a reduction potential of −1.33 V. We assign the first reduction to a reversible cobaltocenium/cobaltocene-Co(III)/Co(II) couple and the second event to the presence of some residual cobaltocenium, proven by additional electrochemical experiments with deliberately added cobaltocenium hexafluoridophosphate that led to an increase of the second reduction observed at approximately −1.42 V.

Upon the first reduction, the color of the solution became a distinctive dark blue, at first indicative of formation of a “ketyl” radical anion. However, simple organic formyl radical anions are unstable due to their insufficient steric protection and dimerize quickly to their corresponding pinacols, whereas ketyl radical anions like the well-known benzophenone radical anion are stable and display an ink blue color which is often used in organometallic chemistry as the so-called “ketyl test” to prove the absence of water and oxygen in anhydrous ether solvents. Furthermore, it is interesting to note that neutral pentamethylcobaltocene carbaldehyde containing Co(II) and five electron-donating methyl groups dimerizes to its pinacol,^6^ in contrast to one-electron-reduced cobaltocenium carbaldehyde hexafluoridophosphate (**3**).

**Scheme 3:**
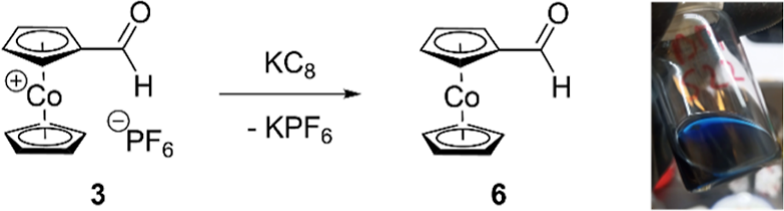
Reduction of Cobaltocenium Carbaldehyde Hexafluoridophosphate (**3**) to Cobaltocene Carbaldehyde (**6**)

Chemical reduction of **3** with one equivalent of potassium graphite was performed under strictly inert conditions in an argon-filled glovebox, and it proved possible to isolate **6** as a dark blue, highly air-sensitive solid (Scheme 3). This material crystallized from a pentane solution at −40 °C, but unfortunately no single crystal structure analysis was possible, because the highly air-sensitive blue crystals dissolved readily at room temperature in the oil used to pick and mount a single crystal.

**Figure 3:**
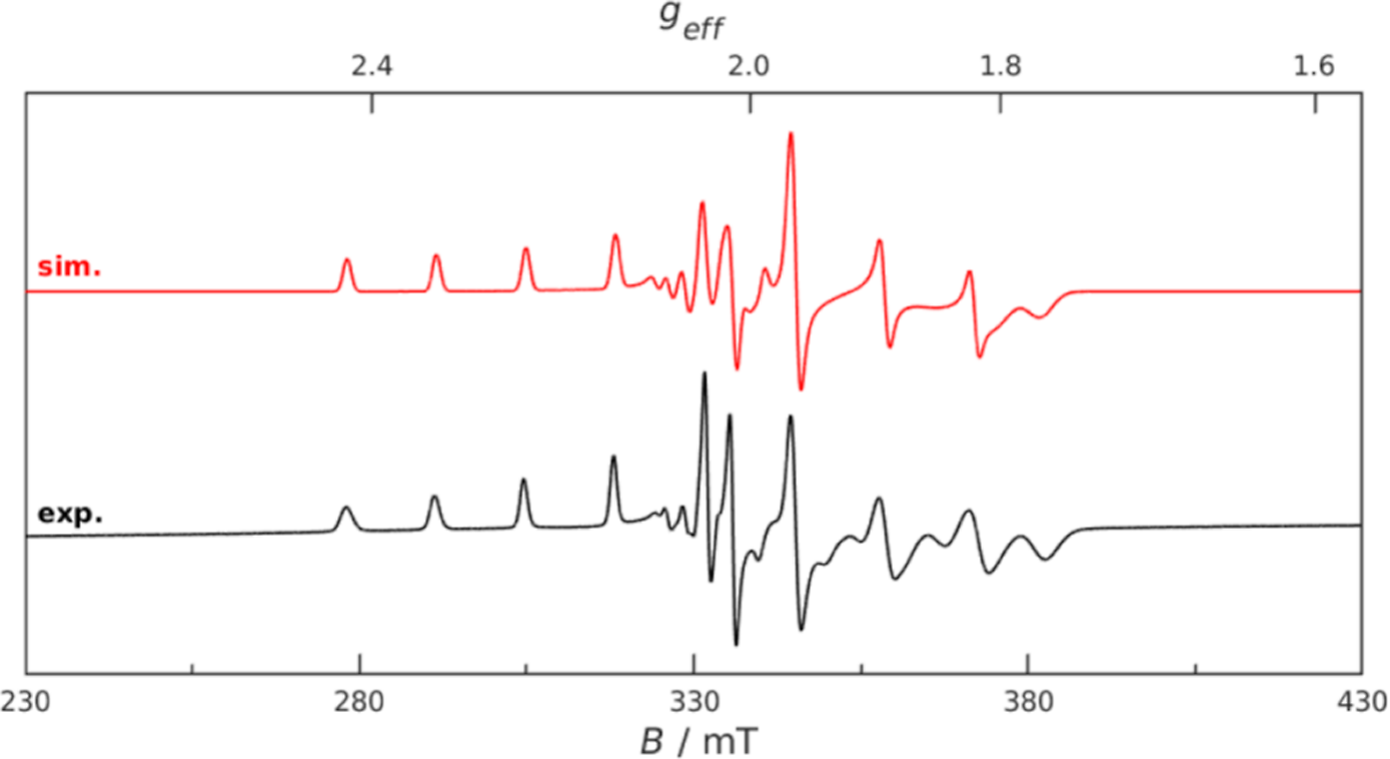
EPR spectrum of cobaltocene carbaldehyde **6** (top: simulated; bottom: in frozen toluene solution at 98 K).

**Figure 4:**
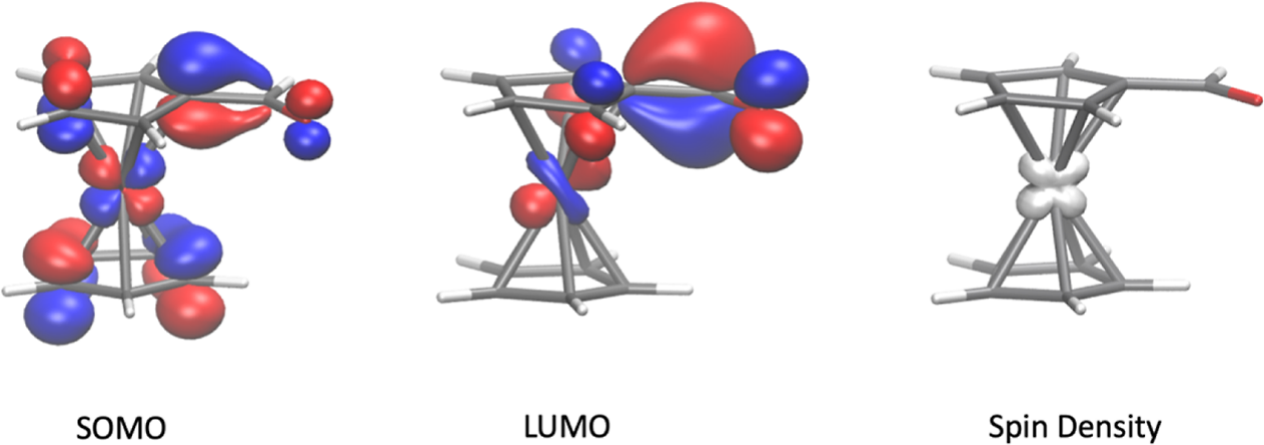
Left: singly occupied canonical molecular orbital (SOMO); middle: lowest unoccupied canonical molecular orbital (LUMO); right: spin density of **6** after optimization with ωB97-xd3/def2-TZVP/CPCM (dichloromethane). An isosurface of 0.05 au is depicted for the molecular orbitals; an isosurface of 0.04 au is depicted for the spin density.

An X-band EPR spectrum of **6** (Figure 3, Table 2) in frozen toluene solution at 98 K showed a well resolved ^59^Co coupling (*I* = 7/2) indicative of the spin density localized fully on the Co(II) center but not on the formyl carbon. This finding is in line with calculated data, where analysis of the spin density indicates that the excess alpha electron is localized at the Co center (Table 2, Figure 4, calculation details in the Supporting Information). Comparable EPR spectra of cobaltocenes containing non-redox-active substituents have been reported in the literature.^16^

**Table 2:** EPR Data for Cobaltocene Carbaldehyde (6) in the Frozen Toluene Matrix at 98 K

*g*_x_	*g*_y_	*g*_z_	*A*_x_	*A*_y_	*A*_z_	method
1.837	2.021	2.082	8.13	85.7	390.5	simulated
1.885	2.040	2.100	3.3	99.3	425.1	calculated^a^

a ωB97XD3(BJ)/aug-cc-pVTZ/CPCM (toluene).

To gain further insights into the (electronic) structure of **6**, density functional theory calculations [ωB97-xd3/def2-TZVP/CPCM (dichloromethane)] were performed. The molecular orbitals (singly occupied molecular orbital: SOMO and lowest unoccupied molecular orbital: LUMO) of **6** (Figure 4) indicate that the SOMO is a metal centered orbital that is delocalized over the entire molecule, while the LUMO is the π* orbital of the CHO group. A Hirshfeld charge analysis shows that the neutral **6** has no significant charges except for the formyl group that has the usual partially positive carbon and partially negative oxygen (details in the Supporting Information). These results were consistent and also found for the double-hybrid B2PLYP density functional (see Supporting Information).

## Conclusions

Cobaltocenium carbaldehyde hexafluoridophosphate, a hitherto elusive monofunctionalized cobaltocenium salt, was synthesized from cobaltocenium carboxylic acid by first converting it to its carboxylic acid chloride followed by reduction with bis(triphenylphosphine)copper(I) tetrahydridoborate to its hydroxymethyl derivative and subsequent Dess–Martin oxidation. Cobaltocenium carbaldehyde hexafluoridophosphate is an untypical aldehyde that shows no standard aldehyde reactivity: no common condensation reaction to Schiff bases were possible; instead, nucleophilic haloform-type cleavage was observed, explainable by the strongly electron-withdrawing cobaltoceniumyl moiety with a formal group electronegativity comparable to that of the pentafluorophenyl group. Cobaltocenium carbaldehyde hexafluoridophosphate is (electro)chemically reversibly reducible to neutral, very air-sensitive, 19-valence-electron formylcobaltocene whose spin-density is only located at the cobalt center but not on the formyl carbon, as shown by EPR spectroscopy and DFT calculations. Therefore, no reductive pinacol coupling is possible, in contrast to not isolable formyl(pentamethyl)cobaltocene which is known to readily form pinacol dimers and other formyl radical follow-up products.

## Experimental Section

### General Procedures

Standard organometallic methods and analytical equipment were used as published previously.^16^ The starting material cobaltocenium carboxylic acid hexafluoridophosphate (**1**) was prepared according to literature.^1i^

Cyclic voltammograms were recorded in a glovebox under an atmosphere of argon, using a BioLogic SP-150 potentiostat with a three-electrode setup (glassy carbon working electrode, platinum wire counter electrode, silver wire pseudo reference) and NBu_4_^+^PF_6_^–^ as supporting electrolyte (0.15 M).^17b,17c,17,17a^ Potentials were calibrated internally to the ferrocene/ferrocenium redox couple.

X-band EPR spectra were obtained on a Bruker Magnettech MS-5000 spectrometer equipped with a temperature controller and using J-Young style quartz glass tubes. Simulations were performed using the pepper function of the EasySpin package^18^ for MatLab.

### Quantum Chemical Calculations

Structures were fully optimized in their respective spin states using the range-separated density functional ωB97-xd3^19a^ in conjunction with the triple-zeta basis set def2-TZVP^19b^ in dicholoromethane, described as a polarizable continuum model with a permittivity of ε = 9.3.^19c^ All calculations were performed with ORCA 5.0.3.^19d^ Molecular orbitals and the spin density were visualized with VMD.^19e^

### Hydroxymethylcobaltocenium Hexafluoridophosphate (**2**)

A Schlenk vessel was charged under an atmosphere of argon with cobaltocenium carboxylic acid hexafluoridophosphate (**1**) (1.04 g, 2.75 mmol, 1 equiv), and 15 mL of thionyl chloride and the mixture was refluxed overnight. SOCl_2_ was evaporated under reduced pressure, and the remaining solids were dried in vacuum. The vessel was transferred into an argon-filled glovebox, and the residue was taken up in 50 mL of dry CH_2_Cl_2_. Triphenylphosphine (866 mg, 3.3 mmol, 1.2 equiv) and bis(triphenylphosphine)copper(I) tetrahydridoborate^10^ (1.82 g, 3.02 mmol, 1.1 equiv) were added in one portion. Gas evolved and the mixture were stirred at ambient temperature overnight. Workup under ambient conditions: the vessel was removed from the glovebox; KPF_6_ (1000 mg, 5.50 mmol; 2 equiv), 25 mL of acetone, and 10 mL of toluene were added. The solvents were reduced to about 10 mL on a rotatory evaporator, 30 mL toluene were added, and the resulting solids were filtered off and washed with Et_2_O and dissolved in CH_3_CN [some white (Ph_3_P)_3_CuCl remains]. For chromatography, approximately 10 g of neutral Al_2_O_3_ was added and CH_3_CN was evaporated on a rotatory evaporator at slightly reduced pressure. A short alumina column (*h* = 3 cm, *Ø* = 4 cm) was first conditioned with a solvent mixture of CH_3_CN/Et_2_O (1:1, v/v) and then dry **2** on Al_2_O_3_ was poured on top. Chromatography was performed using a solvent gradient mixture of 400 mL CH_3_CN/Et_2_O (1:1), followed by 100 mL of CH_3_CN + 5% MeOH, and lastly **2** was eluted with 150 mL of CH_3_CN + 5% MeOH. All volatiles from this fraction were evaporated; some remaining Al_2_O_3_ was separated by dissolving **2** in acetone, filtered, and after evaporation 650 mg of **2** were isolated in 65% yield. **2** is quite air-sensitive and has only limited stability at room temperature; therefore, only characterization by ^1^H NMR was performed: ^1^H NMR (400 MHz, CD_3_CN): δ 5.70–5.68 (m, 2H, Cp_subst_), 5.67 (s, 5H, Cp_unsubst_), 5.61 (t, ^3^*J* = 2.0 Hz, 2H, Cp_subst_), 4.36 (d, ^3^*J* = 5.6 Hz, 2H, CH_2_), 3.66 (t, ^3^*J* = 5.7 Hz, 1H, OH) ppm (see Supporting Information). **2** is immediately used after preparation in the following step.

### Cobaltocenium Carbaldehyde Hexafluoridophosphate (**3**)

A Schlenk vessel was charged with **3** (650 mg, 1.79 mmol, 1 equiv), and 20 mL of dry CH_2_Cl_2_ Dess-Martin periodinane (3-oxo-1λ,^5^ 2-benziodoxole-1,1,1(3*H*)-triyl triacetate) (985 mg, 2,32 mmol, 1.3 equiv) and trifluoroacetic acid (142 μl, 1.79 mmol, 1 equiv) were added; the mixture was stirred at ambient temperature overnight. Workup under ambient conditions: 10 mL CH_3_CN and 4 mL H_2_O were added, and the white solid was filtered off and washed with CH_3_CN. All volatiles were evaporated on a rotary evaporator. The remaining solid was dissolved in 2 mL of CH_3_CN, filtered off, washed with 2 mL of CH_3_CN, reduced to 2 mL, and 50 mL Et_2_O were added for precipitation. After cooling to 4 °C, **3** was filtered off, washed with Et_2_O, and after drying in vacuum, 382 mg of **3** was obtained in 59% yield as yellow powder. **3** is moderately stable in air but should be stored under an inert atmosphere. mp 223.5 °C (dec) ^1^H NMR (300 MHz, (CD_3_)_2_CO): δ 10.18 (s, 1H, CHO), 6.42 (t, ^3^*J* = 2.1 Hz, 2H, Cp_subst_), 6.16 (t, ^3^*J* = 2.1 Hz, 2H, Cp_subst_), 6.05 (s, 5H, Cp_unsubst_) ppm. ^13^C{^1^H} NMR (75 MHz, (CD_3_)_2_CO): δ 189.6 (CHO), 94.99 (C_ipso_), 88.7 (Cp_subst_), 87.2 (Cp_unsubst_), 85.8 (Cp_subst_) ppm. HRMS (ESI+): *m/z* calcd 217.0058 (M^+^); found 217.0055 (M^+^). IR (ATR): 3128 (ν_C–H_), 1702 (ν_C=O_), 1420 (ν_C=C_), 1393, 1373, 1240, 1040, 872, 816 (ν_P–F_), 738, 555 (ν_P–F_), 503, 455, 436 cm^–1^. Suitable single crystals of **3** were obtained from slow evaporation of a solution in a mixture of acetone and MeOH (Figure 1, Supporting Information). Cyclic voltammetry: Figure 2 and Supporting Information. Elemental analysis of **3** was attempted, but values were rather poor; a common problem with cobaltocenium materials.

### Cobaltocene Carbaldehyde (**6**)

In an argon-filled glovebox, **3** (58 mg, 0.16 mmol; 1 equiv) was suspended in 15 mL of dry THF and cooled to −40 °C. Potassium graphite KC_8_ (26 mg, 0.19 mmol; 1.2 equiv) was added, and the suspension was slowly allowed to warm to room temperature under stirring for 2 h. After filtration through a pipet filter, an ink blue solution was obtained. The solvent was removed in vacuum, and the residue was taken up in 5 mL of *n*-pentane and filtered again. **6** was crystallized from this solution as dark blue needle-shaped crystals by reversed diffusion crystallization with toluene at −40 °C. Unfortunately, no single crystal structure analysis proved possible, due to dissolution of **6** at room temperature in the oil used to pick a crystal. EPR spectroscopy (frozen toluene solution, 98 K): EPR data (Figure 3, Table 2). IR and UV–vis data are not available^20^ due to the high air-sensitivity of compound **6**.

## Supporting Information Available

Spectra (^1^H/^13^C NMR, IR, and HRMS), cyclic voltammograms, and DFT computational details (PDF)
Cartesian coordinates of all computed structures (XYZ)

## Supplementary Material

om2c00613_si_001.pdf

om2c00613_si_002.xyz
