# Supplementary material for: Curious Case of Cobaltocenium Carbaldehyde
Source: Organometallics. 2023 Feb 21;42(5):377–83. doi: 10.1021/acs.organomet.2c00613 (PMC10015550; doi:10.1021/acs.organomet.2c00613)
Supplement: Supplementary file 1 — om2c00613_si_001.pdf [file om2c00613_si_001.pdf]

Supporting Information

The Curious Case of Cobaltocenium Carbaldehyde

Daniel Menia,[†] Michael Pittracher,[†] Holger Kopacka, Klaus Wurst, Florian R. Neururer, Daniel Leitner, Stephan Hohloch, Maren Podewitz* and Benno Bildstein*

* maren.podewitz@tuwien.ac.at

* benno.bildstein@uibk.ac.at

[†] These authors contributed equally

1. Spectra

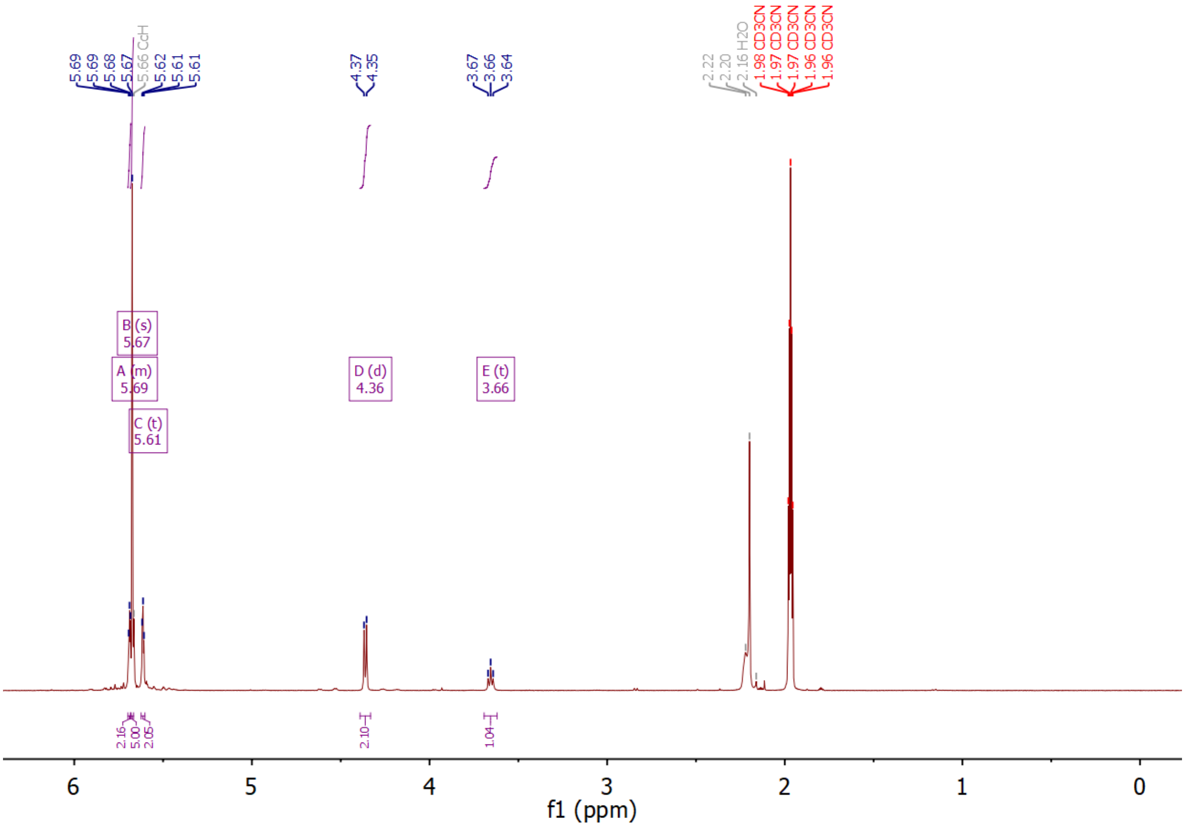

Figure S1: ^{1}H NMR (400 MHz, CD_3CN) **2**.

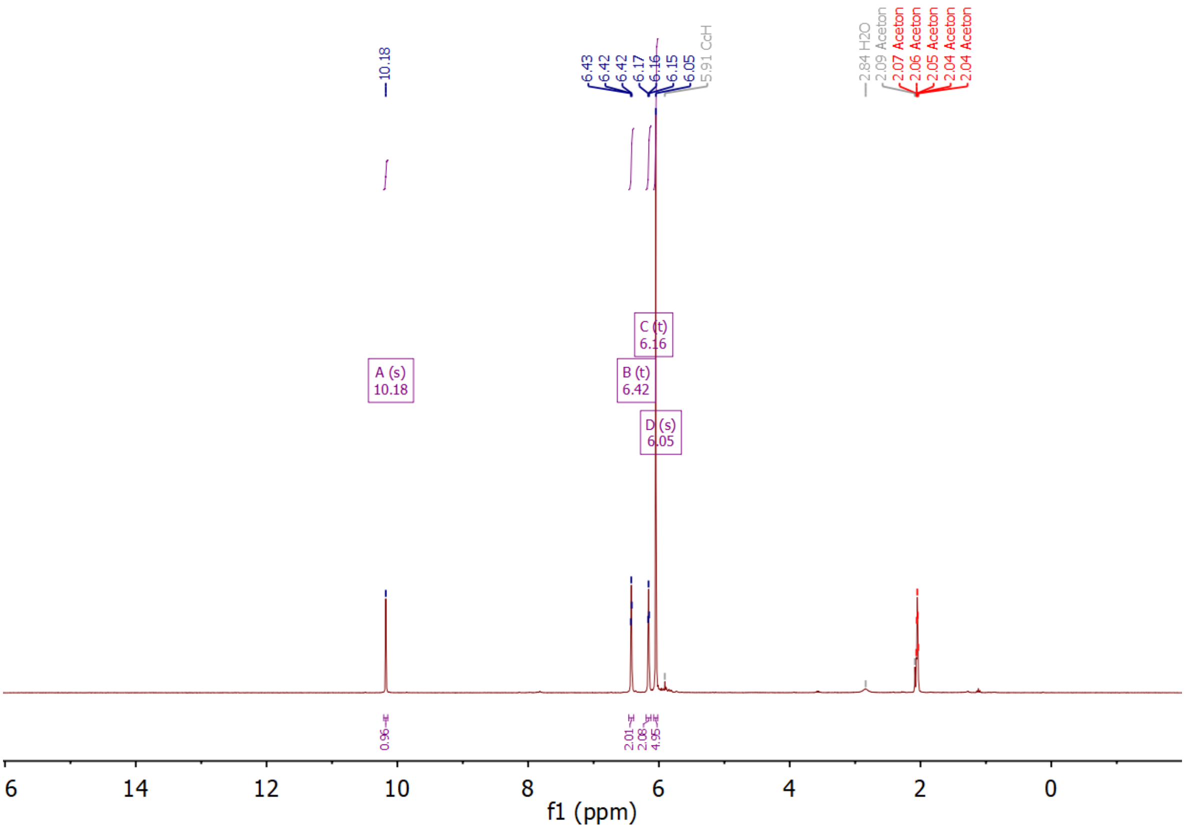

Figure S2: ^{1}H NMR (300 MHz, $(CD_3)_2CO$) **3**.

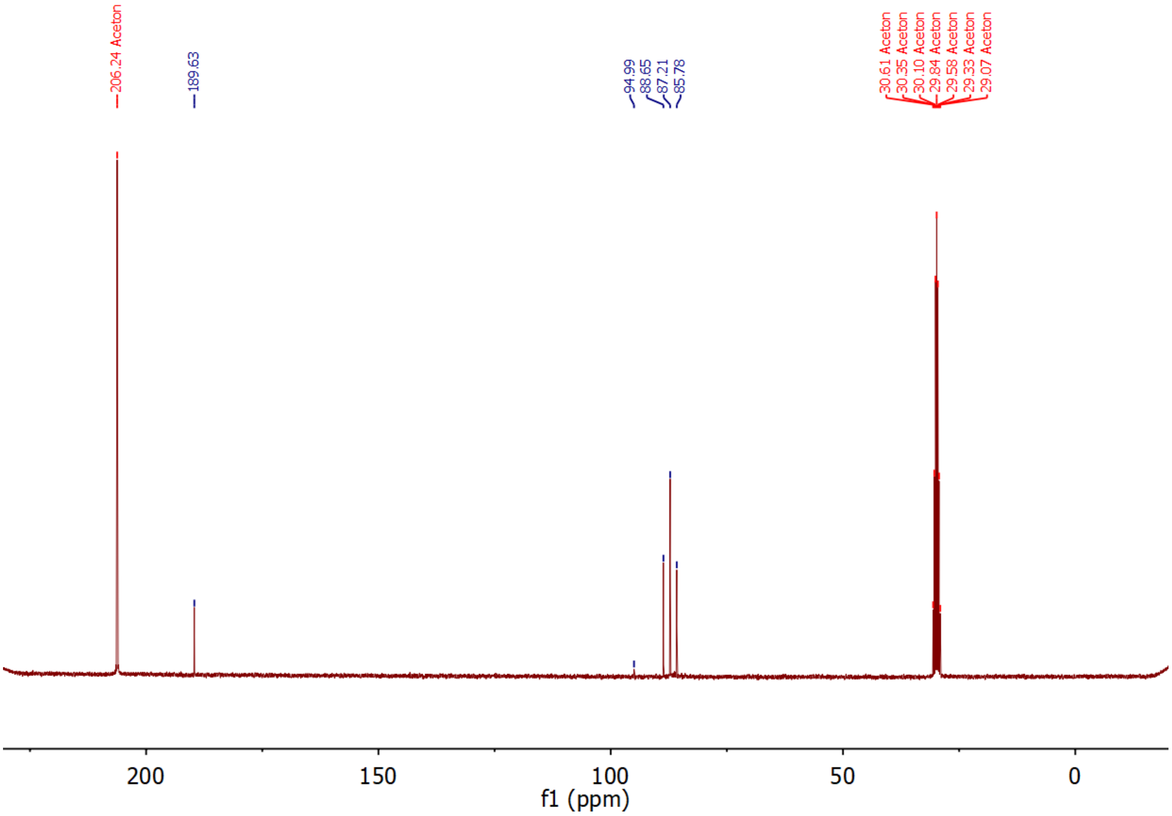

Figure S3: ^{13}C NMR (75 MHz, $(CD_3)_2CO$) **3**.

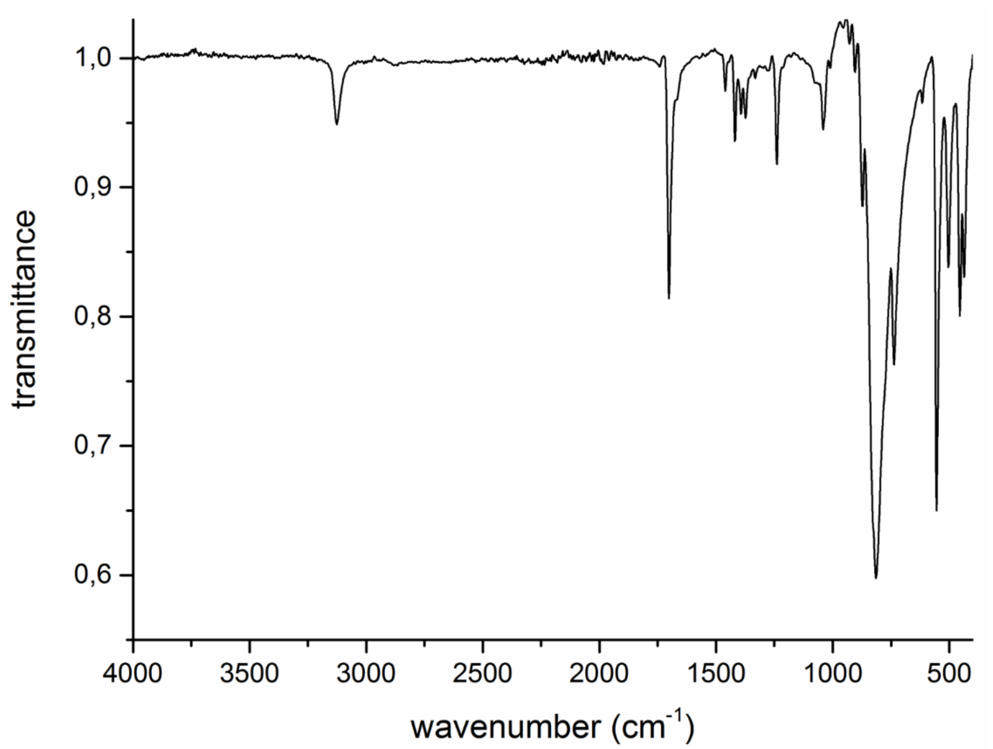

Figure S4: IR (ATR) **3**.

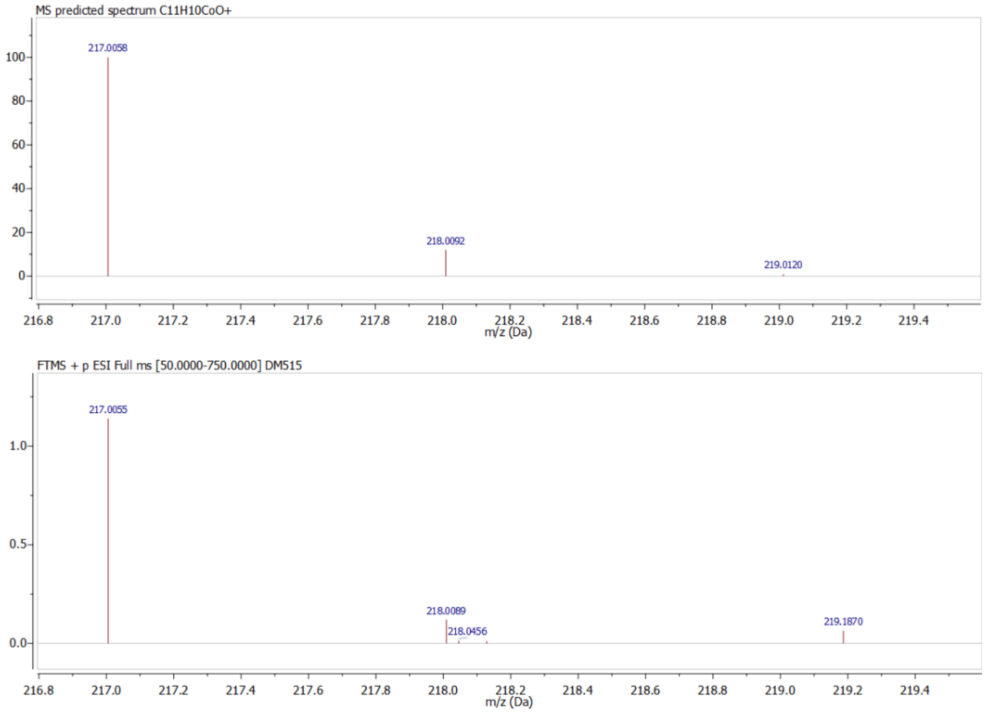

Figure S5: HRMS (ESI+) **3.**

2. Cyclic voltammetry

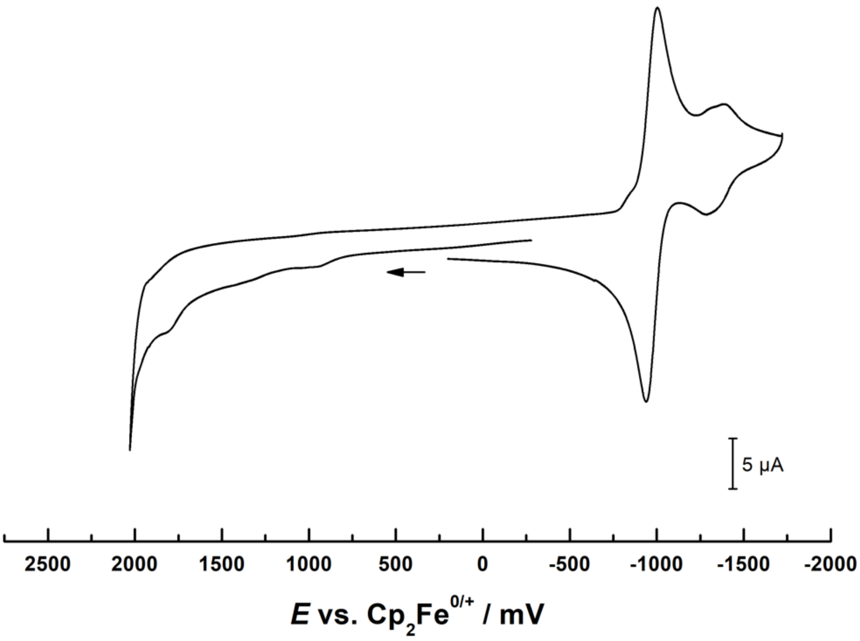

Figure S6: Cyclic voltammogram of **3** at a scan rate of 100 mV/s in CH_3CN/0.15 M NBu_4^+ PF_6^-.

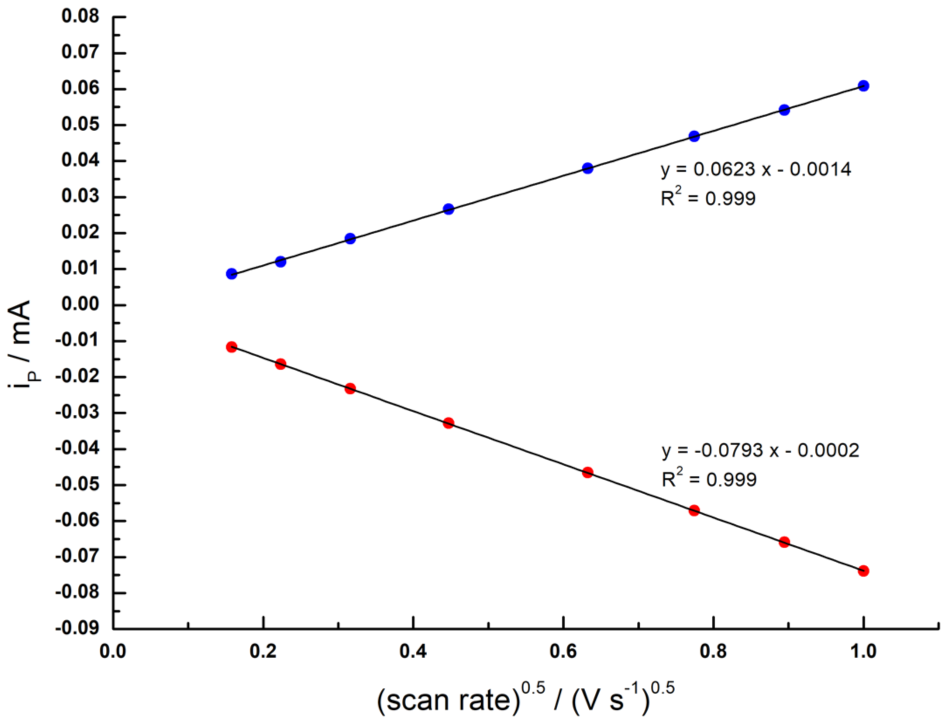

Figure S7: Reversibility plot for the first reduction of **3**.

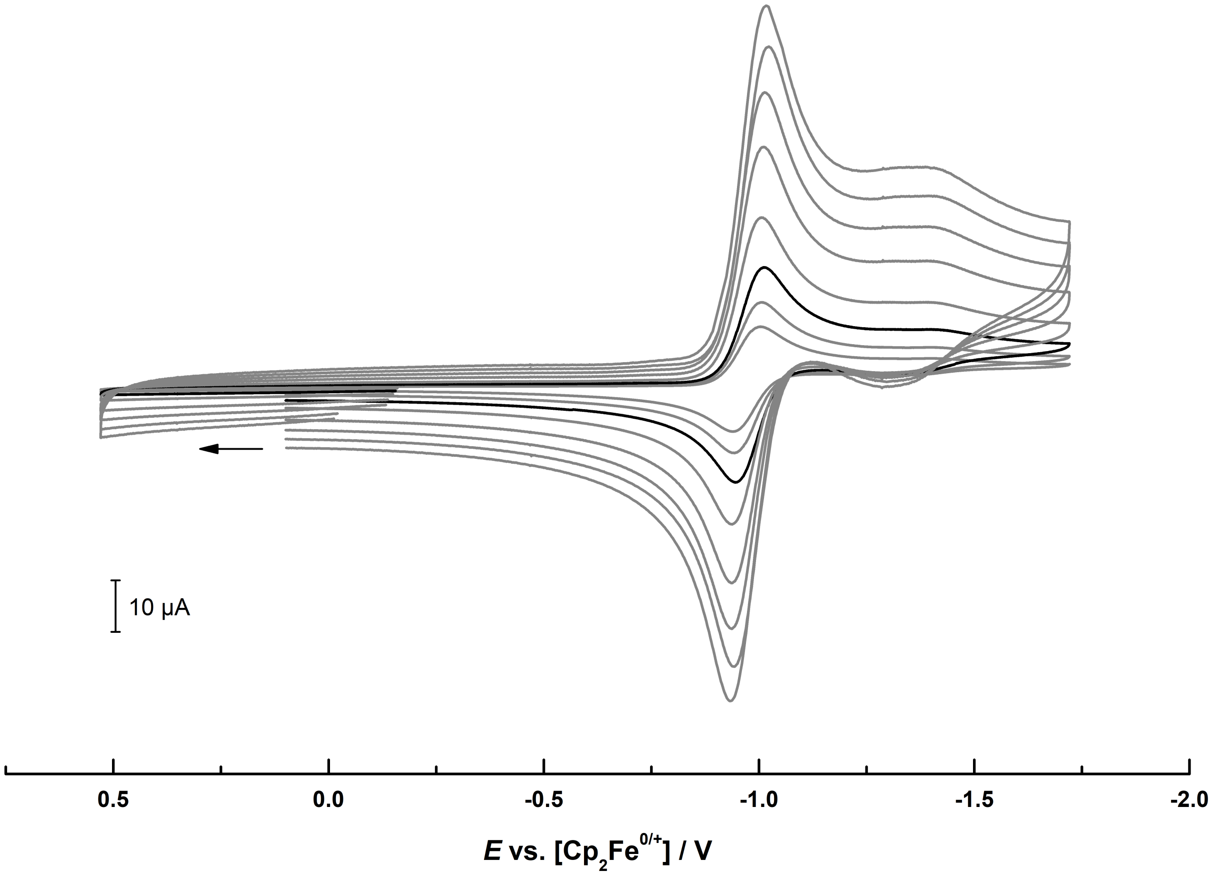

Figure S8: Cyclic voltammogram of **3** at scan rates from 25 mV/s to 1000 mV/s in CH_3CN/0.15 M NBu_4^+ PF_6^-.

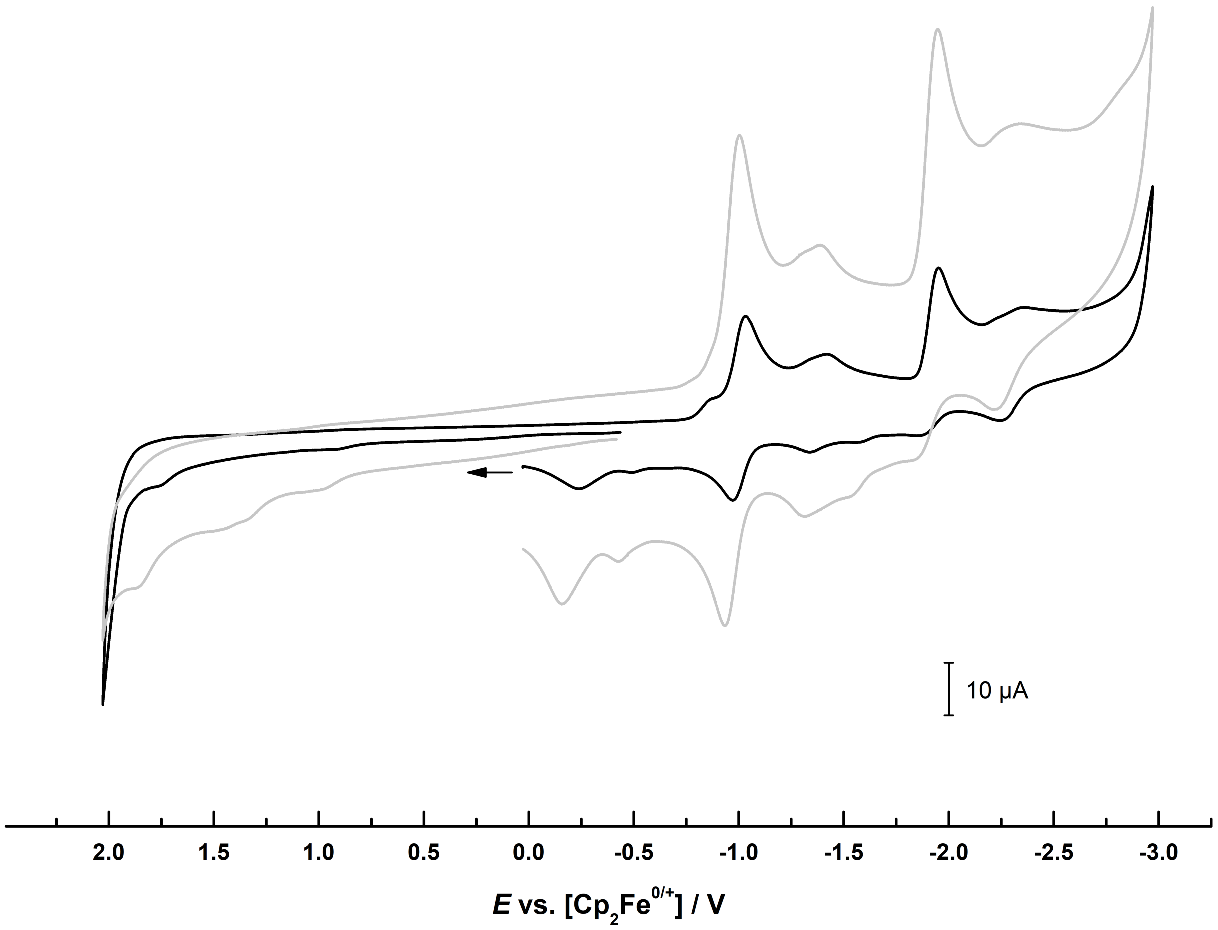

Figure S9: Full range cyclic voltammogram of **3** at scan rates of 0.1 and 0.6 mV/s in CH_3CN/0.15 M NBu_4^+ PF_6^-.

3. DFT Results

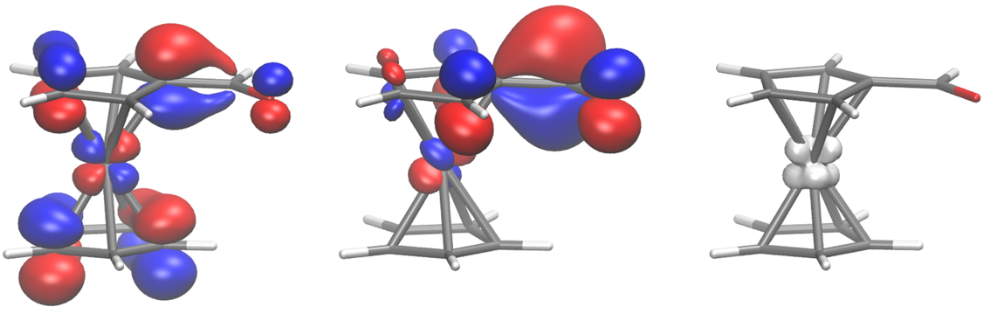

Figure S10: Left: singly occupied canonical molecular orbital (SOMO); middle: lowest unoccupied canonical molecular orbital (LUMO); right: spin density of **6**. All calculation are B2PLYP/def2-TZVP/ CPCM (dichloromethane) single points on the ωB97-xd3/def2-TZVP/ CPCM (dichloromethane) optimized structures. An isosurface of 0.05 a.u. is depicted for the molecular orbitals; an isosurface of 0.04 a.u. is depicted for the spin density.

Table S1. Hirshfeld partial charges as well as spin densities of **6** calculated with the respective methods as indicated. Highlighted in boldface are most notable values.

		ωB97-xd3/def2-TZVP/ CPCM (dichloromethane)		B2PLYP/def2-TZVP/ CPCM (dichloromethane)	
Atom number		Hirshfeld charge	Spin density	Hirshfeld charge	Spin density
1	Co	0.031	**0.841**	0.081	**0.918**
2	C	-0.028	-0.022	-0.031	-0.064
3	H	0.066	-0.002	0.063	-0.004
4	C	-0.038	0.006	-0.037	0.019
5	H	0.063	0.001	0.061	0.002
6	C	-0.036	0.006	-0.035	-0.030
7	H	0.063	0.001	0.061	-0.001
8	C	-0.030	-0.021	-0.032	0.020
9	H	0.062	-0.002	0.060	0.001
10	C	-0.049	0.057	-0.047	0.047
11	C	**0.144**	0.020	**0.160**	0.014
12	H	0.043	0.001	0.045	0.001
13	O	**-0.336**	0.042	**-0.360**	0.040
14	C	-0.054	0.044	-0.054	0.056
15	C	-0.040	-0.022	-0.049	-0.073
16	C	-0.055	0.044	-0.055	0.057
17	C	-0.044	0.001	-0.050	0.002
18	C	-0.046	-0.001	-0.051	-0.010
19	H	0.055	0.004	0.053	0.005
20	H	0.060	-0.002	0.054	-0.005
21	H	0.055	0.004	0.053	0.005
22	H	0.058	0.000	0.054	0.000
23	H	0.058	0.000	0.054	0.000

Calculation Details

Hirshfeld charges

Hirshfeld charges are calculated as a difference between the atomic promolecular electron density and the actual electron density of the molecule.[1]

EPR Data

Electron paramagnetic resonance **g** values were calculated using coupled-perturbed Kohn-Sham DFT as developed by Neese[2] and implemented in ORCA,[3] while the spin-orbit-mean-field method is used to approximate the spin-orbit coupling.[4]

Group Electronegativity (gEN)

To estimate the group electronegativity for Cc^+ and a group of othervarious methods were employed: The first one correlates the natural bond orbital (NBO) charges with (experimental) electronegativity values. Hereby, the NBO charges of the respective group were calculated with B3LYP/aug-cc-PVTZ, were summed up, and plotted against the experimental electronegativity. Linear regression showed a high correlation between the two properties. The corresponding linear equation allowed to determine electronegativity values for unknown species based on calculation of NBO charges.[5]

The second one calculated the electronegativity based on bond polarity indices as proposed by Allen.[6] The bond polarity index is a measure of the difference in the average one electron energy of valence electron of atom A (EI_A) and atom B (EI_B), respectively. To obtain the bond polarity the covalent component had to be removed. To obtain the group electronegativity, EI_A of the corresponding radical has to be calculated, whereas atom A is the atom forms the bond of interest with another fragment. The negative of EI_A is then the gEN. To calculate these gEN values the program multiwfn was used.[7]

As third method, the electronegativity was calculated according to Mulliken as the average of the ionization potential and the electron affinity of the respective group. The ionization potential is the difference between the molecule in its ionized state (*N-1*-electrons) minus its neutral state (*N*-electrons). The electron affinity is the difference between the molecule in its neutral state (*N*-electrons) subtracted by the state, where an electron is attached, (*N+1*-electrons).[8] The neutral species is for each group the radical, e.g., $\cdot CCl_3$ for the $-CCl_3$ group.

References

(1) Hirshfeld, F. L. Bonded-atom fragments for describing molecular charge densities. *Theor. Chim. Acta* **1977**, *44*, 129-138.

(2) Neese, F. Prediction of electron paramagnetic resonance g values using coupled perturbed Hartree–Fock and Kohn–Sham theory. *J. Chem. Phys.* **2001**, *115*, 11080.

(3) (a) Neese, F. Software update: The ORCA program system—Version 5.0. *WIREs ComputMol Sci*. **2022**, *12*, e1606. (b) Neese, F.; Wennemohs, F.; Becker, U.; Riplinger, C. "The ORCA quantum chemistry program package" *J. Chem. Phys.* **2020**, *152*, 224108.

(4) Neese, F. Efficient and accurate approximations to the molecular spin-orbit coupling operator and their use in molecular *g*-tensor calculations. *J. Chem. Phys.* **2005**, *122*, 034107.

(5) (a) Moran, M. D.; Jones, J.-P.; Wilson, A. A.; Houle, S.; Prakash, G. K. S.; Olah, G. A.; Vasdev, N. "Recharging" Group Electronegativities: Computational Chemistry as a Tool for Estimating Electronegativity. *Chem. Educator* **2011**, *16*, 164–167. (b) *Principles and Applications of Quantum*

Chemistry, Gupta, V. R. Academic Press, 2016; chapter 12, Characterization of Chemical reactions, pp 385–433.

(6) Allen, L. C.; Egolf, D. A.; Knight, E. T.; Liang, C. Bond polarity index. *J. Phys. Chem.* **1990**, *94*, 5602-5607.

(7) Lu, T.; Chen, F. Multiwfn: A multifunctional wavefunction analyzer. *J. Comput. Chem.* **2012,** *33*, 580-592.

(8) (a) Mulliken, R. S. A New Electroaffinity Scale; Together with Data on Valence States and on Valence Ionization Potentials and Electron Affinities. *J. Chem. Phys.* **1934**, *2*, 782-793. (b) De Proft, F.; Langenaeker, W.; Geerlings, P. Ab initio determination of substituent constants in a density functional theory formalism: calculation of intrinsic group electronegativity, hardness, and softness. *J. Phys. Chem.* **1993**, *97*, 1826–1831.
